# Host-symbiont co-speciation and reductive genome evolution in gut symbiotic bacteria of acanthosomatid stinkbugs

**DOI:** 10.1186/1741-7007-7-2

**Published:** 2009-01-15

**Authors:** Yoshitomo Kikuchi, Takahiro Hosokawa, Naruo Nikoh, Xian-Ying Meng, Yoichi Kamagata, Takema Fukatsu

**Affiliations:** 1 Institute for Biological Resources and Functions, National Institute of Advanced Industrial Science and Technology (AIST), Tsukuba 305-8566, Japan; 2 Division of Natural Sciences, The Open University of Japan, Chiba 261-8586, Japan; 3 Research Institute of Genome-based Biofactory, National Institute of Advanced Industrial Science and Technology (AIST), Tsukuba 305-8566, Japan

## Abstract

**Background:**

Host-symbiont co-speciation and reductive genome evolution have been commonly observed among obligate endocellular insect symbionts, while such examples have rarely been identified among extracellular ones, the only case reported being from gut symbiotic bacteria of stinkbugs of the family Plataspidae. Considering that gut symbiotic communities are vulnerable to invasion of foreign microbes, gut symbiotic associations have been thought to be evolutionarily not stable. Stinkbugs of the family Acanthosomatidae harbor a bacterial symbiont in the midgut crypts, the lumen of which is completely sealed off from the midgut main tract, thereby retaining the symbiont in the isolated cryptic cavities. We investigated histological, ecological, phylogenetic, and genomic aspects of the unique gut symbiosis of the acanthosomatid stinkbugs.

**Results:**

Phylogenetic analyses showed that the acanthosomatid symbionts constitute a distinct clade in the γ-*Proteobacteria*, whose sister groups are the obligate endocellular symbionts of aphids *Buchnera *and the obligate gut symbionts of plataspid stinkbugs *Ishikawaella*. In addition to the midgut crypts, the symbionts were located in a pair of peculiar lubricating organs associated with the female ovipositor, by which the symbionts are vertically transmitted via egg surface contamination. The symbionts were detected not from ovaries but from deposited eggs, and surface sterilization of eggs resulted in symbiont-free hatchlings. The symbiont-free insects suffered retarded growth, high mortality, and abnormal morphology, suggesting important biological roles of the symbiont for the host insects. The symbiont phylogeny was generally concordant with the host phylogeny, indicating host-symbiont co-speciation over evolutionary time despite the extracellular association. Meanwhile, some local host-symbiont phylogenetic discrepancies were found, suggesting occasional horizontal symbiont transfers across the host lineages. The symbionts exhibited AT-biased nucleotide composition, accelerated molecular evolution, and reduced genome size, as has been observed in obligate endocellular insect symbionts.

**Conclusion:**

Comprehensive studies of the acanthosomatid bacterial symbiosis provide new insights into the genomic evolution of extracellular symbiotic bacteria: host-symbiont co-speciation and drastic genome reduction can occur not only in endocellular symbiotic associations but also in extracellular ones. We suggest that many more such cases might be discovered in future surveys.

## Background

Insects represent the majority of the biodiversity on the earth described, being the most prosperous animal group in the terrestrial ecosystem [1]. One of the key traits considered to make insects so prosperous is their symbiotic association with microorganisms. Symbiotic microorganisms are universally found in the gut, body cavity, or cells of a wide array of insects. Some symbionts are obligate associates for their hosts and significantly contribute to the host fitness, other symbionts are facultative companions of their hosts and tend to negatively affect the host fitness, and the majority of the others are of unknown nature and are recognized only by means of microscopy, PCR detection, and/or DNA sequences. Whether their effects are beneficial, detrimental, or nearly neutral, many of these symbionts substantially affect the physiology, ecology, reproduction, and behavior of their hosts in a variety of ways [2-5].

Among them, the most intimate mutualistic associations are found in obligate endocellular symbionts like *Buchnera *in aphids and *Wigglesworthia *in tsetse flies. In these insects, the symbiotic bacteria are housed in the specialized cells called bacteriocytes or mycetocytes, where the inhabiting symbionts play their physiological roles such as provisioning of essential nutrients for the host insects [6-8]. The symbionts are vertically transmitted to the next host generation in the maternal body at early stages of oogenesis or embryogenesis, wherein the symbiont transmission is integrated into the intricate developmental process of the host insects [2,9]. The symbiont phylogeny usually mirrors the host phylogeny, suggesting host-symbiont co-speciation over evolutionary time [10,11]. In these obligate endocellular bacterial symbionts, remarkable evolutionary patterns, including AT-biased nucleotide composition, accelerated molecular evolution, and reduced genome size, are generally observed in comparison with their free-living relatives. On the basis of these evolutionary patterns, it has been argued that the endocellular lifestyle of the obligate insect symbionts might have strongly affected their genomic evolution [12,13].

On the other hand, symbiotic microorganisms are harbored in the gut cavity of many insects. While most of the gut microbes are commensals or parasites, some of them are known to play substantial biological roles for their hosts. A famous example is wood-eating termites (Isoptera), wherein a rich microbial community of cellulose-degrading bacteria and protozoa in the hindgut is essential for food digestion by the insects [14,15]. Although less known, many plant-sucking stinkbugs (Heteroptera) are also dependent on their gut symbiotic bacteria. In these insects, the terminal region of the midgut is characterized by the presence of many sacs or tubular outgrowths, called crypts or caeca, whose lumen is filled with specific symbiotic bacteria. When experimentally deprived of the microbial associates, the host insects suffer retarded growth, mortality and/or sterility [16-21]. Certainly these gut symbionts are vertically transmitted by superficial bacterial contamination of eggs (egg smearing) or probing parental bacteria-containing excrement (coprophagy) [2,17,18,21,22] and important for their host insects, but researchers have conventionally thought that such extracellular associations are more casual than the endocellular associations, on the grounds that the symbionts are not isolated in the body cavity and thus vulnerable to invasion and replacement by foreign microbes [2,20,23].

However, the conventional view was countered by a recent study on stinkbugs of the family Plataspidae. These insects, which harbor a γ-proteobacterial symbiont named *Ishikawaella capsulata *in their midgut crypts, had been known for their unique mechanism for vertical transmission, so-called 'capsule transmission' [24,25], wherein mother insects deposit symbiont-filled particles (symbiont capsules) in association with eggs, and hatchlings probe the content of the capsules to acquire the symbiont. In the plataspid symbiosis, strict host-symbiont co-cladogenesis and reductive symbiont genome evolution were identified despite the extracellular association [19]. Now it is of evolutionary interest whether similar cases are to be found in other insect-microbe extracellular symbioses or the case of plataspid stinkbugs is an orphan exception.

Here we report a novel group of insect gut bacteria that exhibit host-symbiont co-cladogenesis and reductive genome evolution. The host insects are stinkbugs of the family Acanthosomatidae, which are known for their social behavior such as maternal guarding of eggs and nymphs against predators [26,27]. Rosenkranz [28] described unique histological structures called 'isolated midgut crypts' and 'lubricating organs' for harboring symbiotic bacteria in these insects. In most plant-sucking stinkbugs, the lumen of the midgut crypts is connected to the midgut main tract, and thus the insects are able to excrete the symbiotic bacteria from the anus for vertical transmission to their eggs by surface contamination [2,18,29]. In acathosomatid stinkbugs, by contrast, the lumen of the crypts is completely sealed off, thereby retaining the symbiotic bacteria in the isolated cryptic cavities. The female ovipositor is equipped with a pair of peculiar lubrication organs of unknown developmental origin, which consist of numerous bacteria-filled tubulets. These highly-developed symbiotic organs suggest intimacy of the host-symbiont relationship, but there have been no studies on the acanthosomatid gut symbionts except for the early histological description by Rosenkranz [28].

In this study, we performed comprehensive microbiological characterization of the gut symbiotic bacteria associated with 14 species of acanthosomatid stinkbugs, which unveiled remarkable aspects of their genetic, genomic, and evolutionary features.

## Results

### General observation of midgut crypts

**Figure 1:**
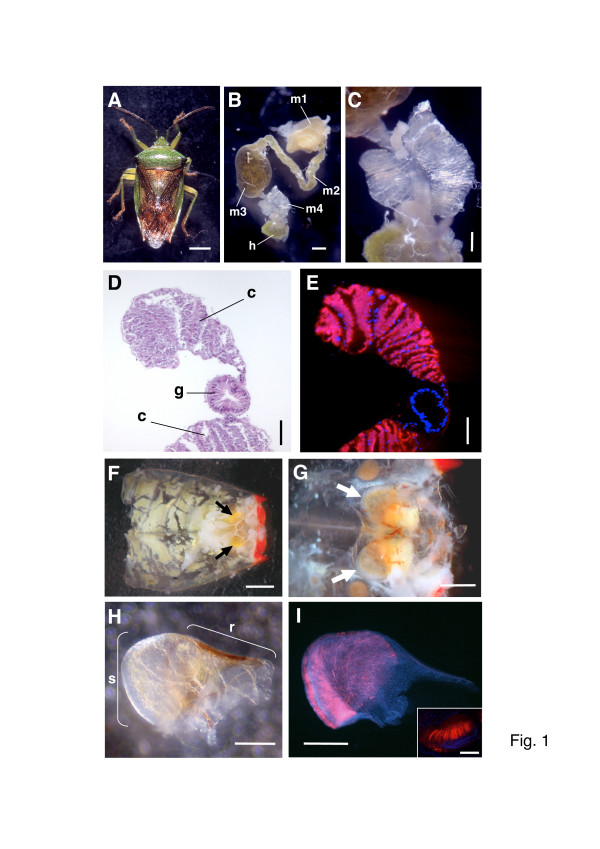
**Specialized organs of *Elasmostethus humeralis *for harboring the symbiotic bacteria**. (A) An adult female. (B) A dissected midgut: m1, midgut first section; m2, midgut second section; m3, midgut third section; m4, midgut fourth section with crypts; h, hindgut. (C) An enlarged image of the midgut fourth section, with butterfly-shaped symbiotic organ consisting of a number of crypts fused two-dimensionally. (D) A sectioned image of the symbiotic organ, stained with hematoxylin and eosin: c, midgut crypt; g, midgut main tract. (E) An *in situ *hybridization image of the symbiotic organ, wherein the symbiotic bacteria (red) and the insect nuclei (blue) are visualized. (F) A dissected ventral abdomen of a female insect, on which a pair of lubricating organs is seen (arrows). (G) An enlarged image of the lubricating organs (arrows) in the posterior tip of the abdomen. (H) A dissected lubricating organ, with the yellow membranous tissue surrounding the organ removed: r, chitinous ridge region; s, sac-like region. (I) An *in situ *hybridization image of the dissected lubricating organ, in which the symbiotic bacteria (red) are specifically detected in the sac-like region. Inset is a confocal image, showing tubulet-like structures harboring the symbiont. Bars, 2 mm in (A), 0.5 mm in (B), 0.2 mm in (C), 100 μm in (D) and (E), 1 mm in (F), 0.5 mm in (G), 0.25 mm in (H) and (I), and 100 μm in (I, inset).

In all of the 14 acanthosomatid species listed in Additional file 1, well-developed crypts were found in the fourth section of the midgut (*Elasmostethus humeralis *and its midgut are shown in Figure 1A–C). The midgut crypts were white in color and arranged in two rows, fused into two-dimensional assemblages and forming a butterfly-shaped organ. When the organs of *E. humeralis*, *Elasmucha signoreti*, *Sastragala esakii*, and *Acanthosoma giganteum *were subjected to sectioning microscopy, no connection was found between the crypt lumen and the midgut main tract (a section image of the midgut crypt of *E. humeralis *is shown in Figure 1D).

### Bacterial 16S rRNA gene sequences from midgut crypts

From 14 acanthosomatid species representing 18 populations (Additional file 1), the crypt-bearing midgut was dissected and subjected to DNA extraction. From the 18 DNA samples, a 1.5 kb segment of bacterial *16S rRNA *gene was amplified by PCR and cloned, and 10 or more clones for each of the samples were subjected to RFLP genotyping. Almost all of the clones derived from a single insect exhibited identical restriction fragment length polymorphism (RFLP) patterns, except for one clone from *E. signoreti *and one from *Acanthosoma labiduroides*.

Three or more clones for each of the 18 samples were sequenced. All of the sequences derived not only from a single insect but also from the same species were completely identical to each other. The sequences from 14 acanthosomatid species showed high sequence similarities ranging from 95.4% to 99.7% to each other. DNA database searches with these sequences identified over 90% similarities to *16S rRNA *gene sequences of γ-proteobacterial representatives. The exceptional clones identified by RFLP genotyping from *E. signoreti *and *A. labiduroides *were also sequenced, which showed the highest similarities to *16S rRNA *gene sequences of *Wolbachia *sp. (supergroup B; M84686; 99.6%) and *Spiroplasma citri *(X63781; 98.1%), respectively.

### *In situ *hybridization of midgut crypts targeting the bacterial 16S rRNA

To confirm whether the obtained *16S rRNA *gene sequences were definitely derived from the gut symbiotic bacteria of the acanthosomatid stinkbugs, we performed 16S rRNA-targeted *in situ *hybridization with specific oligonucleotide probes. In *E. humeralis*, *in situ *hybridization with the specific probes Cy5-EhSym16S and TNKM16S-A555 detected dense signals in the content of the midgut crypts (Figure 1E). Such signals were observed neither in the midgut main tract nor in the tissue connecting the crypts to the main tract. In *Elasmostethus nubilus, S. esakii, E. signoreti*, and *Acanthosoma denticaudum*, *in situ *hybridization with the group-specific probe Cy5-AcSym16S also detected the symbiont signals specifically in the cavity of the midgut crypts in the same manner (data not shown). A series of control experiments confirmed the specificity of the hybridization signals (data not shown).

### Electron microscopy of midgut crypts

**Figure 2:**
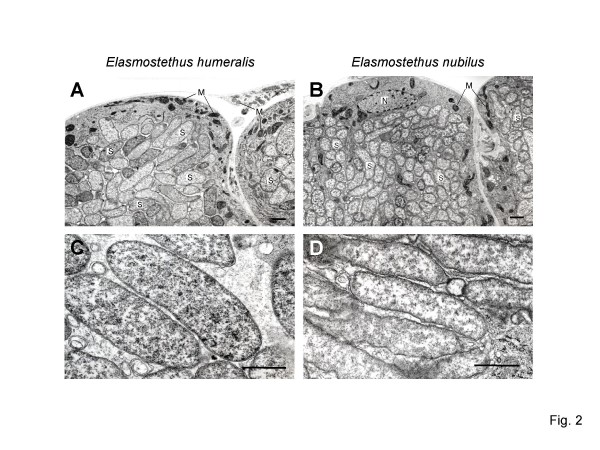
**Transmission electron microscopy of the symbiotic organ of acanthosomatid stinkbugs**. (A) Midgut crypts of *Elasmostethus humeralis*. (B) Midgut crypts of *Elasmostethus nubilus*. (C) Symbiotic bacteria of *E. humeralis*. (D) Symbiotic bacteria of *E. nubilus*. Bars, 1 μm in (A) and (B); 0.3 μm in (C) and (D). Abbreviations: M, mitochondrion; N, nucleus; S, symbiont.

Ultrathin sections of the midgut crypts from *E. humeralis *and *E. nubilus *were observed by electron microscopy. In both the species, the lumen of the crypts was full of symbiotic bacteria (Figure 2A and 2B). The rod-shaped bacterial cells, whose cell wall looked very thin, were larger in *E. humeralis *than in *E. nubilus *(Figure 2C and 2D). The cytoplasm of the epithelial cells of the midgut crypt contained a nucleus and many mitochondria but no bacterial cells (Figure 2A and 2B).

### Phylogenetic analysis of symbiotic bacteria based on *16S rRNA *gene

**Figure 3:**
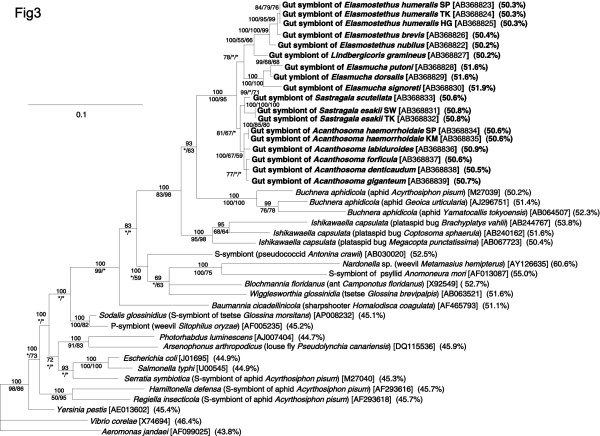
**Phylogenetic placement of the symbiotic bacteria from the acanthosomatid stinkbugs in the γ-*Proteobacteria *on the basis of *16S rRNA *gene sequences**. A total of 1271 aligned nucleotide sites were subjected to the analysis. A Bayesian phylogeny is shown. On the nodes, posterior probabilities in the Bayesian analysis are shown above, and bootstrap probabilities (maximum parsimony (MP) analysis/maximum likelihood (ML) analysis) are shown below. Branches supported by less than 50% posterior probabilities were collapsed into polytomies. Asterisks indicate support values lower than 50%. Sequence accession numbers are in brackets. Percent AT contents of the sequences are in parentheses.

The *16S rRNA *gene sequences originating from the gut symbionts of the acanthosomatid stinkbugs, representing 18 populations, 14 species, and 5 genera, were subjected to molecular phylogenetic analyses together with *16S rRNA *gene sequences of γ-proteobacterial representatives. The acanthosomatid symbionts formed a monophyletic group with high supporting values (100% in Bayesian, 100% in maximum parsimony (MP), and 95% in maximum likelihood (ML), respectively). The phylogenetic relationship of the symbionts was generally in agreement with the systematics of the host insects: the symbionts from congenic host species, *Elasmostethus *spp., *Elasmucha *spp., *Sastragala *spp., and *Acanthosoma *spp., formed clades, respectively. The monophyletic group of the acanthosomatid symbionts showed a phylogenetic affinity to the clade of *Buchnera*, obligate endocellular symbionts of aphids, and also to the clade of *Ishikawaella*, obligate gut symbionts of plataspid stinkbugs (Figure 3).

### Phylogenetic analysis of symbiotic bacteria based on a protein-coding gene

**Figure 4:**
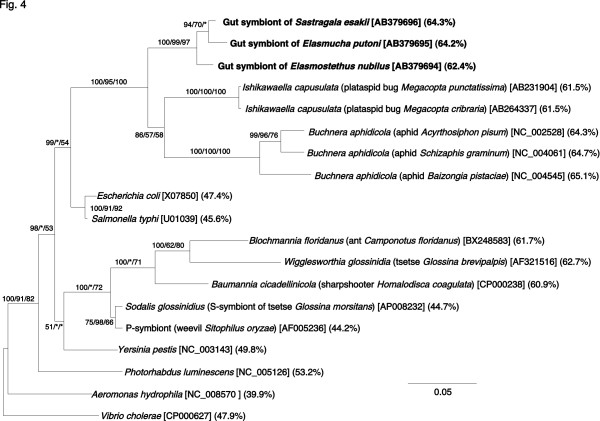
**Phylogenetic placement of the symbiotic bacteria from the acanthosomatid stinkbugs in the γ-*Proteobacteria *on the basis of *groEL *gene sequences**. A total of 1040 aligned nucleotide sites at first and second codon positions were subjected to the analysis. The third codon positions were not used because of saturated nucleotide substitutions. Analysis of deduced amino acid sequences gave substantially the same results (data not shown). A Bayesian tree is shown. Support values for the nodes are indicated as in Figure 3. Asterisks indicate support values lower than 50%. Sequence accession numbers are in brackets. Percent AT contents of the sequences are in parentheses. Note that the AT-content values are based on the data of all codon positions.

To further confirm the phylogenetic placement of the symbiotic bacteria, a *groEL *gene segment was cloned and sequenced from *E. nubilus*, *Elasmucha putoni*, and *S. esakii*. Molecular phylogenetic analyses of the sequences revealed that the sequences of the acanthosomatid symbionts formed a well-supported monophyletic group and clustered with the clade of aphid endosymbionts *Buchnera *and also the clade of plataspid gut symbionts *Ishikawaella *in the γ-*Proteobacteria *(Figure 4). The results of *groEL *gene analyses were generally concordant with the results of *16S rRNA *gene analyses (cf. Figure 3).

### Prevalence of symbiotic bacteria in natural host populations

Diagnostic PCR surveys of field-collected acanthosomatid stinkbugs consistently detected 100% infection frequencies of the symbiotic bacteria in all the species wherein multiple samples were examined: 38/38 in *E. humeralis*; 50/50 in *E. nubilus*; 6/6 in *E. putoni*; and 6/6 in *S. esakii*.

### Detection of symbiotic bacteria from dissected tissues

**Figure 5:**
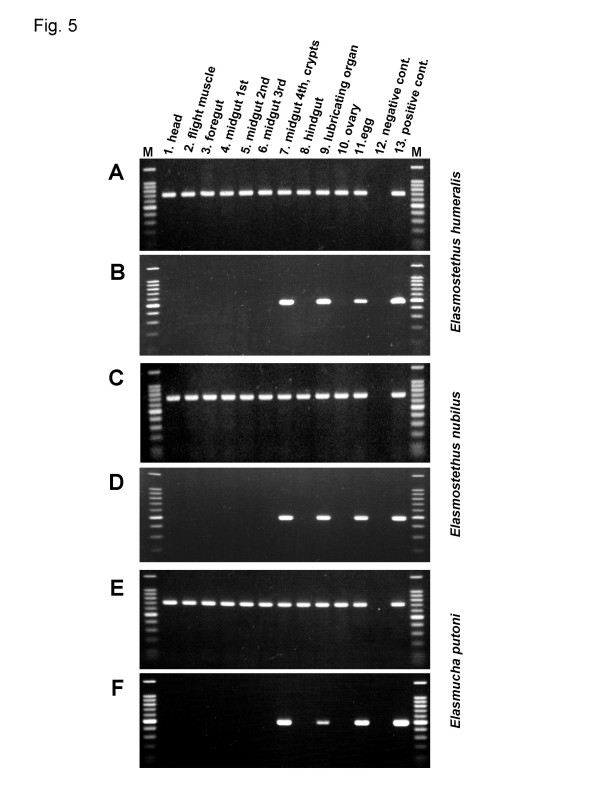
**Diagnostic PCR detection of the symbiotic bacteria from dissected tissues of acanthosomatid stinkbugs**. The results of *Elasmostethus humeralis *(A and B), *Elasmostethus nubilus *(C and D), and *Elasmucha putoni *(E and F) are shown. (A, C, and E) Detection of mitochondrial *COI *gene of the host insects. (B, D, and F) Detection of *16S rRNA *gene of the symbiotic bacteria. Lane 1, head; lane 2, flight muscle; lane 3, foregut; lane 4, midgut first section; lane 5, midgut second section; lane 6, midgut third section; lane 7, midgut fourth section with crypts; lane 8, hindgut; lane 9, abdominal tip containing the lubricating organ; lane 10, ovary; lane 11, egg before hatching; lane 12, no template control; lane 13, positive control (template DNA from midgut crypts of *E. humeralis *TK used for phylogenetic analyses); lane M, DNA size markers (1500, 1000, 900, 800, 700, 600, 500, 400, 300, and 200 bp from top to bottom).

Dissected tissues, including head, flight muscle, foregut, midgut first section, midgut second section, midgut third section, midgut fourth section with crypts, hindgut, abdominal tip, ovary, and egg, were prepared from adult females of *E. humeralis*, *E. nubilus*, and *E. putoni*, and were subjected to diagnostic PCR detection of the symbiotic bacteria. In all the species, positive signals were consistently detected from midgut fourth section, abdominal tip, and egg (Figure 5).

### Vertical transmission of symbiotic bacteria via egg surface contamination

Diagnostic PCR surveys of field-collected egg masses consistently detected the symbiotic bacteria from all the eggs examined: 89 eggs from three egg masses of *E. humeralis*, 23 eggs from one egg mass of *E. nubilus*, 80 eggs from four egg masses of *S. esakii*, and 92 eggs from three egg masses of *A. giganteum *(data not shown). Meanwhile, the symbiotic bacteria were not detected from dissected ovaries of *E. humeralis*, *E. nubilus *and *E. putoni *(Figure 5), refuting the possibility of ovarial symbiont transmission. Newborn nymphs of these species exhibited a characteristic behavior, probing of egg surface with their proboscis, soon after hatching (Kikuchi, personal observation), suggesting the possibility of symbiont transmission via egg surface contamination.

### Sterilization of egg surface disrupted symbiont transmission to newborn nymphs

In an attempt to experimentally confirm the possibility of symbiont transmission via egg surface contamination, we divided each of the egg masses of *E. humeralis *into two portions. One of the halves was left untreated, the other half was surface-sterilized, and newborn nymphs from these experimental egg masses were subjected to diagnostic PCR detection of the symbiotic bacteria after hatching. Most of the nymphs from the control egg masses were symbiont-positive, whereas all of the nymphs from the sterilized egg masses were symbiont-negative (Table 1).

**Table 1:** Effect of surface sterilization of eggs on symbiont acquisition in *Elasmostethus humeralis*.

	Symbiont detection (no. positive/total)	
	
Egg mass number	Control	Surface sterilization ^*a*,*b*^
1	6/6	0/6
2	8/8	0/12
3	15/16	0/18
4	13/13	0/14
5	7/8	0/9
6	6/7	0/7
7	7/7	0/6
8	20/20	0/18

Total	82/85	0/90

^*a*^Eggs were treated with 70% ethanol for 10 min and 4% formaldehyde containing 0.1% Triton X-100 for 30 min, and rinsed two times with 70% ethanol.

^*b*^All samples showed no amplification after 35 cycles of PCR.

### Effects of symbiont elimination on fitness and phenotype of host insects

**Figure 6:**
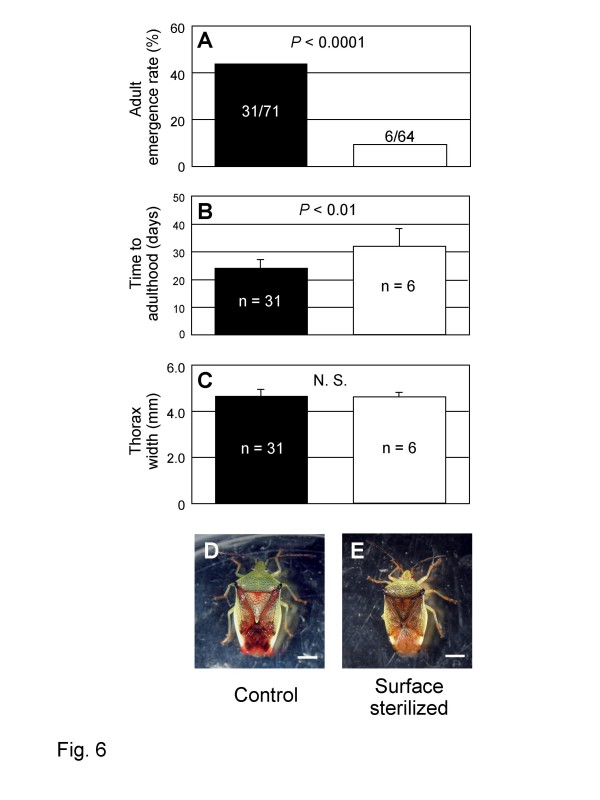
**Effects of symbiont elimination on fitness parameters and phenotype of *Elasmostethus humeralis***. (A) Adult emergence rate (%). Emerged insects per total insects and *P *values of Fisher's exact probability test are indicated. (B) Developmental time to adulthood (days). Means and standard deviations are shown. Sample sizes and *P *values of Mann-Whitney U test are indicated. (C) Body size in terms of thorax width (mm). Means and standard deviations are shown. Sample sizes and *P *values of Mann-Whitney U test are indicated. (D) A normal adult female from a control egg mass. (E) A symbiont-free adult female from a surface-sterilized egg mass, exhibiting abnormal coloration. Bars, 2.5 mm.

Between the control egg masses and the sterilized egg masses, no significant differences were found in time to hatching (control, 4.1 ± 0.5 days, *n *= 182; sterilized, 4.1 ± 0.4 days, *n *= 180) and hatching rate (control, 95.4 ± 6.1%, *n *= 14; surface sterilized, 97.3 ± 4.0%, *n *= 14), indicating that surface sterilization of eggs did not affect the embryonic development of the insect. However, when we examined another set of experimental egg masses of *E. humeralis *for inspection of post-hatch growth and development, drastic differences were detected between the sterilized group and the control group. Adult emergence rate was significantly lower in the sterilized group than in the control group (Figure 6A). Developmental time to adulthood was significantly longer in the sterilized group (31.8 ± 6.6 days, *n *= 6) than in the control group (23.9 ± 2.9 days, *n *= 31) (Figure 6B). Body size in terms of thorax width was not statistically different between the sterilized group (4.59 ± 0.17 mm, *n *= 6) and the control group (4.64 ± 0.32 mm, *n *= 31) (Figure 6C). However, adult insects from the control egg masses were normal in color (Figure 6D), whereas adult insects from the sterilized egg masses exhibited abnormal pale coloration (Figure 6E). Diagnostic PCR detection confirmed that all the adult insects from the sterilized group were symbiont-free, except an individual exhibiting a faint PCR signal (data not shown).

### Localization of symbiotic bacteria in lubricating organ

In the abdomen of adult females of *E. humeralis*, *E. nubilus*, and other acanthosomatid species, we identified a pair of characteristic 'lubricating organs' on the ventral side of the body cavity near the abdominal tip (Figure 1F) which were covered with yellowish membrane (Figure 1G) and lined with cuticular layer (Figure 1H), as previously reported by Rosenkranz [28]. *In situ *hybridization identified with certainty strong signals of the symbiotic bacteria in the lubricating organ (Figure 1I). The symbiont in the lubricating organ was shown to be identical to that in the midgut crypts on the basis of cloning and sequencing of *16S rRNA *gene (data not shown). The lubricating organ consisted of two distinct regions: the sac-like region populated by the symbiotic bacteria and the symbiont-free chitinous ridge region (Figure 1H and 1I). On the outer surface of the sac-like part, numerous tubulet-like structures were densely arranged into a layer, wherein the symbiont signals were localized (Figure 1I, inset). The organ was not found in adult males (data not shown).

### Phylogenetic analysis of host insects based on a mitochondrial gene

**Figure 7:**
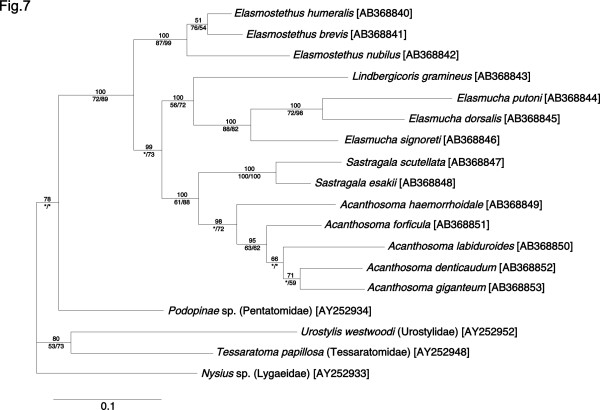
**Phylogenetic relationship of the acanthosomatid stinkbugs on the basis of mitochondrial *COI *gene sequences**. A total of 611 aligned nucleotide sites were subjected to the analysis. A Bayesian phylogeny is shown. Support values for the nodes are indicated as in Figure 3. The phylogeny includes an additional four stinkbug species as outgroup taxa, whose family names are shown in parentheses. Sequence accession numbers are in brackets.

From the 14 acanthosomatid species, a mitochondrial *COI *gene segment was cloned and sequenced. Molecular phylogenetic analyses of the sequences revealed that congenic species, namely *Elasmostethus *spp., *Elasmucha *spp., *Sastragala *spp., and *Acanthosoma *spp., formed distinct clades, respectively (Figure 7), which was in good agreement with the insect systematics.

### Host-symbiont co-evolutionary analysis

**Figure 8:**
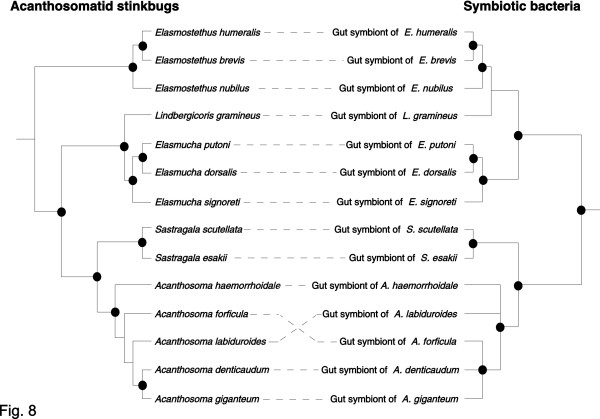
**Phylogenetic congruence between the acanthosomatid stinkbugs and their symbiotic bacteria**. Phylogenetic tree of the host insects (left; cf. Figure 7) and that of the symbiotic bacteria (right; cf. Figure 3) are contrasted. Each symbiont is connected to its host by a dashed line. Dots indicate the 10 co-divergence events inferred by TreeMap.

Figure 8 contrasts the phylogeny of the acanthosomatid stinkbugs and the phylogeny of their gut symbiotic bacteria. The symbiont phylogeny showed a remarkable similarity to the host phylogeny. The only local discrepancies were seen with the placements of *Lindbergicoris gramineus *and *Acanthosoma forficula*/*A. labiduloides*. The history of the host-symbiont association was inferred by two different algorithms using the programs TreeMap [30] and TreeFitter [31]. Both methods identified possible co-evolutionary histories with 10 to 11 co-divergence events (Table 2). The consistently-observed 10 co-divergence events are mapped in Figure 8, based on the result of the TreeMap analysis. Both the TreeMap (Table 2A) and TreeFitter (Table 2B) inferred significantly more co-speciation events than expected from a random distribution, regardless of the chosen topologies for the unsolved nodes. The Icong index [32] also indicated that the topological congruence between the host and symbiont trees was statistically significant (Table 2C).

**Table 2:** Results of the co-divergence analyses with fully resolved trees of the acanthosomatid symbionts.

A. Numbers of the different evolutionary events inferred by TreeMap.								
Symbiont Tree	Reconstruction^*c*^	Codivergences	Duplications	Host switches	Sorting events	Nb of events	Total Cost	P

Max^*a*^	#1	11	2	2	1	16	7.0	< 0.001
Min^*b*^	#1	10	3	3	2	18	11.0	< 0.001
	#2	10	3	3	2	18	11.0	< 0.001

								
B. Mean numbers of the different evolutionary events inferred by TreeFitter.								

Symbiont tree		Codivergences	Duplications	Host switches	Sorting events	Nb of events	Total Cost	P

Max^*a*^		11	0	2	1	14	5.0	< 0.0001
Min^*b*^		10	0	3	2	15	8.0	< 0.0001

								
C. Icong index and significance level.								
					
Symbiont tree		Icong index	P					
					
Max^*a*^		1.84	< 0.0001					
Min^*b*^		1.66	< 0.0005					

^*a*^Tree includes the fully resolved topologies in the symbionts from the genus *Acanthosoma *(topology 2 in Additional file 2), maximizing the host-symbiont congruence.

^*b*^Tree includes the fully resolved topologies in the symbionts from the genus *Acanthosoma *(topology 5 in Additional file 2), minimizing the host-symbiont congruence.

^*c*^Reconstruction with the maximum numbers of co-divergence events and the lowest cost values.

### AT-rich genes of acanthosomatid symbionts

When the base compositions of the *16S rRNA *gene region from γ-proteobacterial representatives were inspected, free-living bacteria like *Escherichia coli *and *Salmonella typhi *exhibited low AT content of around 45%. On the other hand, obligate endocellular insect symbionts such as *Buchnera*, *Wigglesworthia*, *Blochmannia*, and *Baumannia *exhibited remarkably higher AT content of over 50%. The AT content of the acanthosomatid gut symbionts were, together with those of the plataspid gut symbionts *Ishikawaella*, consistently over 50% (cf. Figure 3), which were equivalent to the values of the obligate insect endosymbionts rather than the values of the free-living bacteria. When the base compositions of the *groEL *gene region were examined, similar patterns were observed: free-living bacteria like *E. coli *and *S. typhi *exhibited AT content of around 45%; obligate endocellular insect symbionts such as *Buchnera*, *Wigglesworthia*, *Blochmannia*, and *Baumannia *over 60%; the gut symbionts of plataspid stinkbugs *Ishikawaella *61.5%; and the gut symbionts of acanthosomatid stinkbugs 62–64% (cf. Figure 4).

### Accelerated molecular evolution in acanthosomatid symbionts

Table 3 summarizes the results of relative rate tests for the *16S rRNA *gene from the lineages of acanthosomatid gut symbionts, obligate aphid endosymbionts *Buchnera*, plataspid gut symbionts *Ishikawaella*, and related free-living bacteria. The molecular evolutionary rates in the lineage of acanthosomatid symbionts were significantly higher than those of the free-living bacteria, and were similar to those in the lineages of *Buchnera *and *Ishikawaella*. Table 4 shows the results of relative rate tests for the *groEL *gene sequences, which exhibited similar evolutionary patterns.

**Table 3:** Relative rate tests for comparing the molecular evolutionary rate of *16S rRNA *gene among the lineages of the symbionts of acanthosomatid stinkbugs, symbionts of plataspid stinkbugs, endocellular symbionts of aphids, and their free-living relatives.

Lineage 1^*a*^	Lineage 2^*a*^	Outgroup^*b*^	K1^*c*^	K2^*d*^	Difference of distance^*e*^	Rate ratio^*f*^	*P *value^*g*^
Gut symbionts of acanthosomatid stinkbugs	Gut symbionts of plataspid stinkbugs	S-symbiont of psyllid *Anomoneura mori*	0.068	0.081	-0.013	0.84	0.31
Gut symbionts of acanthosomatid stinkbugs	*Buchnera aphidicola *of aphids	S-symbiont of psyllid *Anomoneura mori*	0.065	0.093	-0.028	0.70	0.17
Gut symbionts of plataspid stinkbugs	*Buchnera aphidicola *of aphids	S-symbiont of psyllid *Anomoneura mori*	0.094	0.109	-0.015	0.86	0.30
Gut symbionts of acanthosomatid stinkbugs	*Escherichia coli *and *Salmonella typhi*	*Yersinia pestis*	0.103	0.033	0.070	3.1	< 0.0001
Gut symbionts of plataspid stinkbugs	*Escherichia coli *and *Salmonella typhi*	*Yersinia pestis*	0.163	0.030	0.133	5.4	< 0.0001
*Buchnera aphidicola *of aphids	*Escherichia coli *and *Salmonella typhi*	*Yersinia pestis*	0.187	0.029	0.158	6.4	< 0.0001

^*a*^Acanthosomatid symbionts from *E. nubilus *(AB368822), *E. humeralis *(AB368823, AB368824, AB368825), *E. brevis *(AB368826), *L. gramineus *(AB368827), *E. putoni *(AB368828), *E. dorsalis *(AB368829), *E. signoreti *(AB368830), *S. esakii *(AB368831, AB368832), *S. scutellata *(AB368833), *A. haemorrhoidale *(AB368834, AB368835), *A. labiduroides *(AB368836), *A. forficula *(AB368837), *A. denticaudum *(AB368838), *A. giganteum *(AB368839). Plataspid symbionts from *Megacopta punctatissima *(AB067723), *Brachyplatys subaeneus *(AB244767), and *Coptosoma sphaerula *(AB240162). Aphid symbionts from *Geoica urticularia *(AJ296751), *Yamatocallis tokyoensis *(AB064507) and *Acyrthosiphon pisum *(M27039). *E. coli *(J01695) and *Salmonella typhi *(U00545).

^*b*^S-symbiont of psyllid *A. mori *(AF013087); *Y. pestis *(AE013602).

^*c*^Estimated mean distance between lineage 1 and the last common ancestor of lineages 1 and 2.

^*d*^Estimated mean distance between lineage 2 and the last common ancestor of lineages 1 and 2.

^*e*^K1–K2.

^*f*^K1/K2.

^*g*^*P *value based on the null distribution of distance values generated by 10,000 bootstrap resamplings of the aligned nucleotide sites.

**Table 4:** Relative rate tests for comparing the molecular evolutionary rate of *groEL *gene (nucleotide 1st, 2nd positions of codon) among the lineages of the symbionts of acanthosomatid stinkbugs, symbionts of plataspid stinkbugs, endocellular symbionts of aphids, and their free-living relatives.

Lineage 1^*a*^	Lineage 2^*a*^	Outgroup^*b*^	K1^*c*^	K2^*d*^	Difference of distance^*e*^	Rate ratio^*f*^	*P *value^*g*^
Gut symbionts of acanthosomatid stinkbugs	Gut symbionts of plataspid stinkbugs	Closely related endosymbionts	0.034	0.065	-0.031	0.52	0.0170
Gut symbionts of acanthosomatid stinkbugs	*Buchnera aphidicola *of aphids	Closely related endosymbionts	0.036	0.096	-0.060	0.38	0.0002
Gut symbionts of plataspid stinkbugs	*Buchnera aphidicola *of aphids	Closely related endosymbionts	0.056	0.084	-0.028	0.67	0.0884
Gut symbionts of acanthosomatid stinkbugs	*Escherichia coli *and *Salmonella typhi*	*Vibrio cholerae*	0.090	0.018	0.072	5.0	< 0.0001
Gut symbionts of plataspid stinkbugs	*Escherichia coli *and *Salmonella typhi*	*Vibrio cholerae*	0.111	0.011	0.100	10.1	< 0.0001
*Buchnera aphidicola *of aphids	*Escherichia coli *and *Salmonella typhi*	*Vibrio cholerae*	0.177	0.0	0.177	N.D.^*h*^	< 0.0001

^*a*^Acanthosomatid symbionts from *E. nubilus *(AB379694), *E. putoni *(AB379695), *S. esakii *(AB379696). Plataspid symbionts from *Megacopta punctatissima *(AB231904) and *Megacopta cribraria *(AB264337). Aphid symbionts from *Acyrthosiphon pisum *(NC_002528), *Schizaphis graminum *(NC_004061), *Baizongia pistaciae *(NC_004545) and *Cinara cedri *(NC_008513). *E. coli *(X07850) and *Salmonella typhi *(U01039).

^*b*^Closely related endosymbionts from *Wigglesworthia glossinidia *from tsetse *Glossina brevipalpis *(AF321516), *Blochmannia floridanus *from ant *Camponotus floridanus *(BX248583), *Baumannia cicadellinicola *from sharpshooter *Homalodisca coagulata *(CP000238). *Vibrio cholerae *(AE013602).

^*c*^Estimated mean distance between lineage 1 and the last common ancestor of lineages 1 and 2.

^*d*^Estimated mean distance between lineage 2 and the last common ancestor of lineages 1 and 2.

^*e*^K1–K2.

^*f*^K1/K2.

^*g*^*P *value based on the null distribution of distance values generated by 10,000 bootstrap resamplings of the aligned nucleotide sites.

^*h*^Divided by 0.

### Reduced genome size of acanthosomatid symbionts

**Figure 9:**
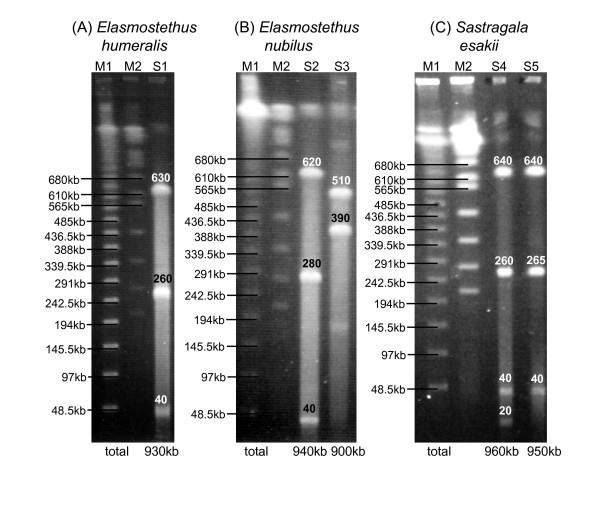
**Pulsed field gel electrophoresis of the symbiont genomic DNA prepared from dissected midgut crypts of the acanthosomatid stinkbugs**. (A) *Elasmostethus humeralis*. (B) *Elasmostethus nubilus*. (C) *Sastragala esakii*. Lane M1, size marker (*Saccharomyces cerevisiae *chromosomes); lane M2, size marker (lambda ladder); lane S1, *I-CeuI *digestion for *E. humeralis*; lane S2, *I-CeuI *digestion for *E. nubilus*; lane S3, *AscI *digestion for *E. nubilus*; lane S4,*ApaI *digestion for *S. esakii*; lane S5, *SmaI *digestion for *S. esakii*. Sizes of genomic DNA fragments are depicted on the gel images. Estimated genome sizes are shown under the gel images.

Figure 9 shows the pulsed-field gel electrophoresis of the genomic DNA of the acanthosomatid gut symbionts. The symbiont genome sizes were estimated to be 0.93 Mb for *E. humeralis*, 0.90–0.94 Mb for *E. nubilus*, and 0.95–0.96 Mb for *S. esakii*. The genome sizes were much smaller than those of free-living γ-proteobacteria like *E. coli *(4.6 Mb), *S. typhi *(4.8 Mb), and *V. cholerae *(4.0 Mb), and close to those of obligate endocellular insect symbionts such as *Buchnera *(0.42–0.65 Mb), *Wigglesworthia *(0.70 Mb), *Blochmannia *(0.71–0.79 Mb), and *Baumannia *(0.69 Mb), and also to those of plataspid gut symbionts *Ishikawaella *(0.82–0.83 Mb).

## Discussion

### Identification of novel gut symbiotic bacteria from acanthosomatid stinkbugs

This study provides the first genetic and evolutionary characterization of the symbiotic bacteria from stinkbugs of the family Acanthosomatidae since the early histological description by Rosenkranz [28]. All of the 14 species representing 5 genera that we examined possessed a well-developed symbiotic organ, consisting of a number of closed tubular crypts fused two-dimensionally, in the midgut fourth section (Figure 1). Electron microscopy showed that rod-shaped bacterial cells, which are morphologically uniform with reduced cell wall, are packed in the cavity of the crypts extracellularly (Figure 2). Genotyping and sequencing of bacterial *16S rRNA *gene revealed that a specific γ-proteobacterium dominates the microflora in the symbiotic organ of each of the stinkbug species. Molecular phylogenetic analyses based on the *16S rRNA *gene and *groEL *gene sequences demonstrated that these bacteria from the acanthosomatid stinkbugs form a monophyletic group in the γ-*Proteobacteria *(Figures 3 and 4). Diagnostic PCR and *in situ *hybridization confirmed with certainty that the *16S rRNA *gene sequences originate from the symbiotic bacteria in the isolated cavity of the midgut crypts (Figures 1 and 5). On the basis of these results, we conclude that the acanthosomatid stinkbugs harbor a specific clade of γ-proteobacteria as extracellular symbiont in the highly-specialized midgut crypts.

### Relationship to gut symbiotic bacteria of other stinkbugs

A diverse array of stinkbugs are associated with gut symbiotic bacteria, most of which are harbored in the lumen of the midgut crypts [2,23,33]. Despite these extensive histological descriptions, only a limited number of stinkbug gut symbionts have been microbiologically characterized, including γ-proteobacterial *Ishikawaella *from stinkbugs of the family Plataspidae [19], β-proteobacterial *Burkholderia *from broad-headed bugs of the family Alydidae [34], nocardioform actinomycetes from assassin bugs of the family Reduviidae [35], and an unnamed γ-proteobacterium from the southern green stinkbug *Nezara viridula *of the family Pentatomidae [22]. Molecular phylogenetic analyses indicated that the clade of the acanthosomatid gut symbionts is related to but distinct from the clade of the plataspid gut symbionts *Ishikawaella *in the γ-*Proteobacteria *(Figures 3 and 4). These results indicate that the bacterial symbiont characterized in this study is specifically associated with the family Acanthosomatidae, and strongly suggest that symbiotic associations with diverse microbes have evolved repeatedly and independently in different stinkbug lineages.

### Host-symbiont co-speciation in acanthosomatid stinkbugs

In obligate endocellular symbiotic bacteria of diverse insects, such as *Buchnera *in aphids, *Wigglesworthia *in tsetse flies, *Blochmannia *in ants, *Baumannia *in sharpshooters, *Carsonella *in psyllids, *Portiera *in whiteflies, *Nardonella *in weevils, and others, the symbiont phylogeny generally mirrors the host phylogeny, indicating stable and intimate host-symbiont associations over evolutionary time [10,11,36-40]. By contrast, in gut symbiotic bacteria of various insects, such as those in termites and alydid stinkbugs, the symbiont phylogeny does not reflect the host phylogeny, suggesting promiscuous host-symbiont associations over evolutionary time [34,41,42]. Conventionally, it has been thought that such extracellular symbiotic associations are evolutionarily more casual than the endocellular symbiotic associations, on the grounds that the symbionts are not isolated in the body cavity and vulnerable to invasion and replacement by foreign microbes [2]. Recently, however, strict host-symbiont co-speciation was discovered in the gut symbiotic bacteria *Ishikawaella *of plataspid stinkbugs [19]. In this study, we unveiled another example of co-speciating gut symbiotic bacteria in acanthosomatid stinkbugs (Figure 8), corroborating the idea that host-symbiont co-speciation can occur even in gut symbiotic associations. The co-cladogenetic pattern suggests that a single bacterial infection in the common ancestor of the Acanthosomatidae has been stably maintained over evolutionary time, diversifying in parallel with the host speciation. The stable host-symbiont association is suggestive of important biological roles of the symbiont for the host insect and strict vertical transmission of the symbiont through host generations.

### Vertical symbiont transmission via egg surface contamination

From eggs of acanthosomatid species, the symbiotic bacteria were consistently detected, whereas dissected ovaries contained no symbiotic bacteria (Figure 5). Newborn nymphs of these species exhibited a characteristic behavior, probing of egg surface with their proboscis, soon after hatching (Kikuchi, personal observation). Sterilization of egg surface resulted in disruption of symbiont transmission to newborn nymphs of *E. humeralis *(Table 1). These results indicate that the symbiont is vertically transmitted via egg surface contamination. In addition to the Acanthosomatidae, this type of symbiont transmission has been also known from the family Pentatomidae [21,22,28], whereas different types of symbiont transmission have been reported from other groups of stinkbugs, including probing of parental bacteria-containing excrement (coprophagy) from the families Cydnidae and Coreidae [17,18], deposition of bacteria-containing capsules with eggs (capsule transmission) from the family Plataspidae [16,19,43], and acquisition of free-living bacteria from the environment every generation (environmental acquisition) from the family Alydidae [20]. Probably because of the extracellular symbiont location in the gut cavity, these stinkbugs have evolved post-hatch symbiont transmission mechanisms instead of the ovarial transmission mechanisms typical of endocellular symbionts of various insects.

### Symbiont-dependent host growth and development

Thus far, diverse stinkbugs from the families Pentatomidae, Cydnidae, Plataspidae, Coreidae, Alydidae etc have been reported to suffer retarded growth, sterility and/or mortality when experimentally deprived of their gut symbiotic bacteria [16-21]. In this study, we provided the first experimental demonstration that the gut symbiont is important for normal growth and development of *E. humeralis*, a member of the family Acanthosomatidae: when deprived of the symbiont, the insects exhibited lower adult emergence rate, prolonged developmental time, and abnormal morphology (Figure 6). The biological roles played by the symbiont are currently unknown. Acanthosomatid stinkbugs suck leaves, stems, and fruits of their host plants [44]. Plausibly, the symbiont may provide the host with nutritional supplements, such as essential amino acids and vitamins, as has been demonstrated in other plant-sucking insects [3,8].

### Novel symbiotic organ 'isolated midgut crypts'

In most of the stinkbug groups, a number of sac- or tube-like crypts are present in a posterior region of the midgut, and the cavity of the crypts, wherein the symbiotic bacteria are harbored, has a connection to the midgut main tract [2,23,33]. Hence, the midgut main tract constantly receives symbiont supply from the crypts, which can be used for vertical transmission of the symbiont by egg surface contamination or other means. In the Acanthosomatidae, however, the cavity of the crypts is completely disconnected from the midgut main tract (Figure 1) [28]. The isolated midgut crypts form a highly-specialized symbiotic organ, in which the symbiotic bacteria are confined not in the cytoplasm but in the extracellular space (Figure 2). It is expected that the isolated cavity enables stable cultivation of the symbiotic bacteria that perform essential biological roles for the host insect. How the symbiotic organ is formed deserves future study. Probably the midgut crypts are not closed in newborn nymphs, the symbiotic bacteria ingested by the nymphs colonize the rudimentary crypts, and the cryptic cavity is disconnected from the midgut main tract in the developmental course of the insects. Rosenkranz [28] observed that the midgut crypts are connected to the main tract at least until the second nymphal instar in *Acanthosoma haemorrhoidale*.

### Novel transmission apparatus 'lubricating organ'

While suitable for stable retention of the symbiotic bacteria, the isolated midgut crypts in the Acanthosomatidae entail a serious problem: symbiont supply to anal excrement for vertical transmission is, unlike the other stinkbug groups, structurally disrupted. The dilemma is, astonishingly, reconciled by the evolution of a novel 'lubricating organ' associated with the female ovipositor. Rosenkranz [28] reported that, on the ventral side of the posterior abdomen, adult females possess a pair of sac-like organs with a chitinous lining consisting of numerous bacteria-filled tubulets. Considering the structural configuration, it was postulated that the organ is arranged in such a way that eggs gliding downwards in the ovipositor squeeze out a portion of the bacteria-containing fluid into the vagina, whereby the egg surface is contaminated. In this study, our histological observations confirmed the description by Rosenkranz [28] (Figure 1E–H). *In situ *hybridization and diagnostic PCR demonstrated the microbiological identity of the bacteria in the isolated midgut crypts, those in the lubricating organ, and those on the egg surface (Figures 1 and 5). However, the development, infection process and evolutionary origin of the lubricating organ are not yet fully understood, deserving detailed histological and developmental studies in future.

### Occasional lateral transfer of acanthosomatid symbiont: possible relevant factors

Despite the global host-symbiont co-cladogenesis, it was inferred that host switching of the symbiotic bacteria might have occurred at least twice in the acanthosomatid stinkbugs (Figure 8). How such lateral symbiont transfers have taken place in the acanthosomatid species is currently unknown, but it may be relevant that the postnatal symbiont transmission via egg smearing is potentially vulnerable to horizontal symbiont transmission between host lineages [34]. Here, although speculative, we suggest the possibility that plant utilization patterns of the acanthosomatid stinkbugs might have been involved in the process. For example, a number of acathosomatid species such as *E. nubilus, S. esakii, S. scutellata, A. labiduroides*, and *A. forticula *commonly feed and reproduce on the same plant, *Swida controversa *[44]. Under the condition that egg-laying females of different species coexist on the same plant, wandering newborn nymphs might encounter an opportunity to acquire the symbiotic bacteria from heterospecific egg masses. On the other hand, we suggest the possibility that the maternal brood care behavior typical of the stinkbug group might have countered the process. In acanthosomatid species such as *E. putoni, E. dorsalis, E. signoreti*, *S. esakii*, *S. scutellata*, and *A. gigantium*, adult females lay an egg mass on the host plant, and guard the eggs and first instar offspring against predators by a series of defensive behaviors, including covering the clutch with the body, tilting the body toward the source of disturbance, strongly fanning the wings, etc. [45,46]. These anti-predator behaviors might also discourage the access of wandering newborn nymphs to heterospecific egg masses, thereby preventing the lateral symbiont transfer. In these contexts, experimental studies on lateral symbiont transfers between different acanthosomatid stinkbugs are of ecological and evolutionary interest.

### Reductive genome evolution in acanthosomatid symbiont

In most obligate endocellular symbiotic bacteria like *Buchnera *in aphids, *Wigglesworthia *in tsetse flies, *Blochmannia *in ants, *Baumannia *in sharpshooters, and others, remarkable evolutionary patterns, including AT-biased nucleotide composition, accelerated molecular evolution, and reduced genome size, have been detected in comparison with their free-living relatives [6,7,47-51]. These peculiar genetic traits have been argued in relation to the attenuated purifying selection due to small population size and strong bottleneck, which are associated with the lifestyle of vertically transmitted endocellular symbionts [12,13]. Here it should be noted that small population size and strong bottleneck are also found in vertically transmitted extracellular symbionts, like some gut symbiotic bacteria of stinkbugs. Recently, from the gut symbiotic bacteria of plataspid stinkbugs *Ishikawaella*, AT-biased genes, accelerated evolution, and genome reduction were identified [19]. In this study, we demonstrated that the gut symbiotic bacteria of acanthosomatid stinkbugs also exhibit these peculiar genetic traits (Figures 3, 4, and 9; Tables 3 and 4). These findings suggest that the reductive genome evolution in the insect symbionts is not simply attributable to their endocellular or extracellular habitats. Anyway, stable living inside the host insect symbiotically may relax natural selection acting on a number of biological processes that are needed only for non-symbiotic life, leading to degeneration and loss of genes relevant to those processes over evolutionary time. Also, it appears plausible that attenuated purifying selection due to small population size and strong bottleneck might have facilitated the reductive genome evolution.

### Proposal of candidate name

On account of the remarkable and distinct genetic, genomic, and microbiological traits described above, we propose the designation '*Candidatus *Rosenkranzia clausaccus' for the symbiotic bacteria of the acanthosomatid stinkbugs.

#### (i) Diagnostic features

The symbiont belongs to the γ-*Proteobacteria *(Figures 3 and 4). The alignment of *16S rRNA *gene sequences from 18 isolates of the symbiont, representing 5 genera and 14 species of the host insects, plus other proteobacteria indicates the following distinctive residues (numbering based on the *E. coli *sequence): GAA at positions 141 to 143, GAGGAAGAAA at positions 444 to 453, AUGA(U/C)A at positions 472 to 477, and AGG(U/C)UGU(A/G)AGC(U/A)UGACUU at positions 831 to 848. The *16S rRNA *gene sequences reported here are AB368822-AB368839. Also available are *groEL *gene sequences from three isolates of the symbiont, AB379694-AB379696.

The symbiont is harbored in the extracellular cavity of the symbiotic organ, consisting of a number of closed tubular crypts fused two-dimensionally, in the terminal region of the midgut (Figure 1B–E; Figure 5). In adult females, the symbiont is also found in the lubricating organ associated with the ovipositor (Figure 1F–I; Figure 5). Electron microscopy shows that the symbiont is a rod-shaped bacterium, whose size and shape differ between the host species (Figure 2). The symbiont is vertically transmitted by egg surface contamination with symbiont-containing secretion from the lubricating organ associated with the female ovipositor (Table 1; Figure 5). The symbiont genes exhibit high AT content: 50.2–51.9% for *16S rRNA *gene (Figure 3) and 62.3–64.1% for *groEL *gene (Figure 4). The symbiont genes exhibit accelerated molecular evolution (Tables 3 and 4). The symbiont genome size is 0.90–0.96 Mb (Figure 8).

#### (ii) Hosts

The symbiont is associated with stinkbugs of the family Acanthosomatidae, including *E. humeralis, E. nubilus, Elasmostethus brevis*, *L. gramineus, E. putoni, Elasmucha dorsalis, E. signoreti, S. esakii, S. scutellata, A. labiduroides, A. denticaudum, A. forficula, A. giganteum*, and *A. haemorrhoidale *(Additional file 1). Early histological descriptions [28] suggest that the symbiont is also found in other acanthosomatid species including *Elasmostethus interstinctus, Elasmostethus minor, Elasmucha ferrugata, Elasmucha fieberi, Elasmucha grisea, Cyphostethus tristiatus, Planois bimaculata*, and *Ditomotarsus gayi*, although no molecular data are available for them. The host phylogeny is largely concordant with the symbiont phylogeny (Figure 7), suggesting stable host-symbiont association over evolutionary time. Elimination of the symbiont results in retarded growth, elevated mortality, and abnormal coloration of the host insects (Figure 6). The symbiont has not been cultured outside the host insects.

#### (iii) Nomenclature

The generic name honors Werner Rosenkranz, who first described the symbiotic system of the acanthosomatid stinkbugs [28]. The specific name refers to the completely isolated midgut crypts for harboring the symbiotic bacteria (*clausus *= closed, *saccus *= bag or sack).

## Conclusion

Our findings presented in this study strongly suggest that the endocellular or extracellular environment matters little in the symbiont genome evolution. Symbiotic lifestyle itself, which entails relaxed natural selection, small population size, and strong bottleneck, is likely to have substantially affected the reduced symbiont genomes. Here, in a broader evolutionary context, we call attention to the point that such population genetic features are not restricted to symbiotic bacteria of insects and other organisms, but may be generally found in bacterial parasites and pathogens intimately associated with their host organisms. For obligate bacterial parasites and pathogens that suffer no or inferior survival outside their eukaryotic hosts including insects, animals, and even humans, the microbial populations are expected to be more or less divided and structured, potentially leading to small effective population size and strong bottleneck. It should be noted that similar reductive genome evolution is also observed in bacterial pathogens like *Rickettsia*, *Chlamydia*, and *Mycoplasma*, among which some are endocellular and others are not [52-54]. Hence, the same evolutionary logic operating in the obligate endocellular and extracellular bacterial symbionts may also apply to the bacterial parasites and pathogens, illuminating the evolutionary continuum from parasitism through commensalism to mutualism.

Whilst the obligate endocellular and extracellular insect symbionts exhibit globally similar patterns of reductive genome evolution, we expect that close examination of their genomes would reveal notable differences reflecting their adaptation to and constraint in the different symbiotic lifestyles. In this context, it may be notable that the genome sizes of the extracellular symbionts, *Ishikawaella *and *Rosenkranzia*, are somewhat larger than those of endocellular ones like *Buchnera *and *Wigglesworthia*. The phylogenetic affinity of the aphid endocellular symbionts *Buchnera*, the plataspid gut symbionts *Ishikawaella*, and the acanthosomatid gut symbionts *Rosenkranzia *(Figures 3 and 4) provides an ideal opportunity for comparative genomic analyses, which would lead to further insights into how their symbiotic lifestyles have affected their molecular, evolutionary, and genomic features.

## Methods

### Insect materials

Acanthosomatid stinkbugs representing 5 genera, 14 species, and 20 populations were examined in this study (Additional file 1). Adult females were mainly used, whereas egg masses collected in the field were subjected to diagnostic PCR analyses. The samples were preserved in acetone or 99% ethanol immediately after collection until molecular and histological analyses were carried out [55]. For electron microscopy, adult insects were brought alive to the laboratory, dissected, and fixed. Egg masses of *E. humeralis *deposited by field-collected adult females in the laboratory were used for experiments of symbiont elimination and fitness measurement.

### DNA extraction

Dissected insect tissues and isolated eggs were individually subjected to DNA extraction using the NucleoSpin Tissue Kit (MACHEREY-NAGEL). For tissue preparation, the preserved insects were softened by soaking in 70% ethanol, and dissected under a binocular microscope by using fine forceps. From each of the adult females, head, flight muscle, foregut, swollen midgut first section, thin midgut second section, swollen midgut third section, midgut fourth section with crypts, hindgut, ovary, and abdominal tip containing the lubricating organs were dissected.

### Cloning, genotyping and sequencing

A 1.5 kb segment of bacterial *16S rRNA *gene was amplified with the primers 16SA1 (5'-AGA GTT TGA TCM TGG CTC AG-3') and 16SB1 (5'-TAC GGY TAC CTT GTT ACG ACT T-3') [56]. A 1.7 kb segment of bacterial *groEL *gene was amplified with the primers Gro-F1 (5'-ATG GCA GCW AAA GAC GTA AAT TYG G-3') and Gro-R1 (5'-TTA CAT CAT KCC GCC CAT GC-3') [57]. A 0.6 kb segment of insect mitochondrial *COI *gene was amplified with the primers LCO1490 (5'-GGT CAA CAA ATC ATA AAG ATA TTG G-3') and HCO2198 (5'-TAA ACT TCA GGG TGA CCA AAA AAT CA-3') [58]. PCR was conducted by using Ampli*Taq*Gold DNA polymerase (Applied Biosystems) and its supplemented buffer system under a temperature profile of 95°C for 10 min followed by 30 cycles of 95°C for 30 sec, 55°C (*16S rRNA *gene), 55°C (*groEL *gene) or 48°C (*COI *gene) for 1 min, and 72°C for 1 min. The PCR products were cloned with TA-cloning vector pT7Blue (Takara) and *E. coli *DH5α competent cells (Takara). Inserted clones were screened by direct colony PCR with the primers Univ19 (5'-GTT TTC CCA GTC ACG ACG T-3') and Rev20 (5'-AGC TAT GAC CAT GAT TAC GC-3') that flank the cloning site of the vector. As for *16S rRNA *gene, 10 or more amplified products from each of the samples were subjected to restriction fragment length polymorphism (RFLP) genotyping by using restriction endonucleases *HaeIII *and *RsaI*. Three or more plasmid clones from each of the samples were prepared by using QIAprep-Spin Miniprep Kit (QIAGEN). The purified plasmids were eluted with 50 μl of distilled water, and subjected to DNA sequencing by using BigDye Terminator Cycle sequencing Kit (Applied Biosystems) and ABI PRISM 377 DNA sequencer (Perkin Elmer) as previously described [34].

### Histology

The preserved insects were dissected in phosphate buffered saline (PBS: 137 mM NaCl, 8.1 mM Na_2_HPO_4_, 2.7 mM KCl, 1.5 mM KH_2_PO_4 _[pH 7.5]), and the dissected tissues were transferred to Carnoy's solution (ethanol:chloroform:acetic acid = 6:3:1). After an overnight fixation, the tissues were treated with 6% hydrogen peroxide in 80% ethanol for three days for quenching autofluorescence [59], and then kept in 100% ethanol at room temperature. The samples were cleared through an ethanol-xylene series, embedded in paraffin, and processed into 5 μm serial sections on a rotary microtome. The tissue sections were mounted on silane-coated glass slides, dewaxed through a xylene-ethanol series, and air-dried prior to hematoxylin-eosin staining or *in situ *hybridization. For whole-mount *in situ *hybridization, the preserved samples were washed twice in PBS and directly subjected to hybridization procedures.

### *In situ *hybridization

The following fluorochrome-conjugated probes for detection of *16S rRNA *of the symbiotic bacteria were used for *in situ *hybridization: Cy5-EhSym16S (5'-Cy5-CAT CCT TAT TAA GAA TGA TCG-3') specifically targeting the symbiont of *E. humeralis*; TNKM16S-A555 (5'-Alexa555-CCT TCA TTA GGC AGA TCC-3') and Cy5-AcSym16S (5'-Cy5-TTT GTA TAC ACC ATT GTA-3') targeting the acanthosomatid symbionts collectively.*In situ *hybridization was performed essentially as described previously [34]. The tissue samples were incubated in a hybridization buffer (20 mM Tris-HCl [pH 8.0], 0.9 M NaCl, 0.01% SDS, 30% formamide) containing 50 nM of either of the probes. For counter-staining of host insect nuclei, 4 μM of 4',6-diamidino-2-phenylindole (Invitrogen) or 0.5 μM of SYTOX Green (Invitrogen) was added to the hybridization buffer. After an overnight incubation, the samples were thoroughly washed in PBS and mounted in Slowfade antifade solution (Molecular Probes). The sectioned and whole-mount samples were observed under an epifluorescent microscope (Axiophot, Carl Zeiss) and a laser scanning confocal microscope (LSCM Pascal5, Carl Zeiss), respectively. To confirm specificity of the detection, a series of control experiments were conducted as previously described [59].

### Electron microscopy

The tissues containing the midgut crypts were dissected from live insects in 2.5% glutaraldehyde in 0.1 M sodium cacodylate buffer (pH 7.4), prefixed in the fixative at 4°C overnight, and postfixed in 2% osmium tetroxide at 4°C for 60 min. After dehydration through an ethanol series, the materials were embedded in Spurr resin (Nisshin-EM). Ultrathin sections (thickness, 80 nm) were made on an ultramicrotome (Ultracat-N, Leichert-Nissei), mounted on collodion-coated copper meshes, stained with uranyl acetate and lead citrate, and observed under a transmission electron microscope (model H-7000, Hitachi).

### Molecular phylogenetic analysis

Multiple alignments of the nucleotide sequences were generated using the program CLUSTAL W [60]. The alignments were then inspected and corrected manually, from which ambiguously aligned sites were removed. Phylogenetic analyses were conducted by the three methods maximum parsimony (MP), maximum likelihood (ML) and Bayesian. MP and ML trees were constructed using the program PAUP 4.0b10 [61]. In the MP analysis, all sites and character changes were weighted equally. In the ML analysis, we selected the GTR + I + G model for the *16S rRNA*, *groEL *and *COI *gene phylogenies, on the basis of the Akaike criterion estimated by the program Modeltest 3.06 [62]. Bootstrap tests were performed with 100 replications in the MP and ML analyses. In the Bayesian analysis, the programs MrBayes v3.0b4 [63] and MrModeltest v2.1 [64] were used. The Akaike criterion selected the GTR + I + G model for the *16S rRNA*, *groEL *and *COI *gene phylogenies. In total 8000, 10 000 and 4500 trees were obtained for the *16S rRNA *gene (ngen = 120 000, samplefreq = 10, burn in = 4000), the *groEL *gene (ngen = 160 000, samplefreq = 10, burn in = 6000) and the host *COI *gene (ngen = 60 000, samplefreq = 10, burn in = 1500), respectively. Based on the data, a 50% majority-rule consensus tree was constructed.

### Co-evolutionary analysis

Based on the host and symbiont Bayesian trees, the history of host-symbiont association was investigated by the programs TreeMap v2.02β [30,65] and TreeFitter v1.0 [31], and host-symbiont co-divergence level was evaluated by randomization tests. Although TreeMap and TreeFitter require fully resolved trees, the symbiont phylogeny contained several unsolved parts, particularly in the genus *Acanthosoma *(cf. Figure 3). Thus, of the nine possible fully-resolved patterns of the symbiont phylogeny (cf. Additional file 2), two trees were *a priori *chosen and inspected: one was maximizing the co-divergence number and the other minimizing it. TreeMap uses a model to find optimal reconstructions of the association history by maximizing co-divergence events [30], while TreeFitter minimizes total event costs to find optimal reconstructions [31]. In both the programs, reconciliation analyses were performed under the default setting of the event costs (TreeMap: co-divergence = 0; duplication = 1; host switch = 1; valuesorting = 1; TreeFitter: co-divergence = 0; duplication = 0; host switch = 2; sorting = 1). Then, the extent of the observed host-symbiont co-evolution was statistically evaluated by a randomization test. A distribution of expected score, the number of co-divergence events for TreeMap and global cost for TreeFitter, was produced by comparing randomly generated symbiont trees and the observed host tree, on which statistical significance of the observed score was tested. In TreeMap and TreeFitter, 1000 and 10,000 symbiont trees were randomly generated, respectively.

Phylogenetic congruence between host and symbiont was also evaluated by using Icong index [32], wherein topological congruence of two trees is assessed through their maximum agreement subtrees (MAST). A MAST is the largest possible tree compatible with two given trees and is obtained by removing the minimum number of leaves (terminal branches) in both trees to obtain perfect congruence. Significance of topological congruence was inferred by a randomization test concerning the size of the MAST.

### Relative rate test

Genetic distances were estimated under the HKY substitution model and the gamma distribution of rate variation across nucleotide sites by using the program TREE-PUZZLE5.2 [66]. To take into account the phylogenetic relationship among the sequences, the genetic distances between the lineages were calculated as weighted average [67]. The significance of the rate differences was evaluated by generating 10 000 bootstrap replicates of the alignment sites [68]. In these tests, we excluded all aligned sites containing a gap or an unresolved base in any of the sequences considered.

### Diagnostic PCR

A 0.5 kb segment of *16S rRNA *gene of the symbiotic bacteria of acanthosomatid stinkbugs was amplified with the primers 16SA1 and TNKM16SR (5'-GYG AGT AAC GTC AAT TAT CAT NCT-3') under a temperature profile of 95°C for 10 min followed by 30 cycles of 95°C for 30 sec, 55°C for 1 min and 72°C for 1 min. To confirm the quality of the DNA samples, a segment of mitochondrial *COI *gene was amplified with the primers LCO1490 and HCO2198 as described above.

### Surface sterilization of eggs

Each egg mass of *E. humeralis *was divided into two portions. One was untreated, whereas the other was treated with 70% ethanol for 10 min, 4% formaldehyde containing 0.01% Triton X-100 for 30 min, and rinsed thoroughly with 70% ethanol twice. Each of the experimental egg masses was kept in a Petri dish with a wet cotton ball at 25°C until hatching. To confirm successful elimination of the symbiont, hatchlings from the experimental egg masses were subjected to DNA extraction and diagnostic PCR two days after the second instar molt.

### Measurement of fitness parameters

A pair of adult female and male *E. humeralis *was reared in a plastic case (8 cm in diameter, 5.5 cm in depth), and fed with fruits of udo plant (*Aralia cordata*) and dried seeds of peanut (*Arachis hypogaea*) at 25°C under a long day regimen (16 h light, 8 h dark). Egg masses laid by four pairs of the insect were used for the following experiments. The control and sterilized egg mass portions were examined for time to hatching and hatching rate. The hatchlings from the experimental egg mass portions were separately reared as described above. Growth and survival of the nymphs were monitored until all of them either reached adulthood or died. The following parameters were recorded: adult emergence rate, time to adulthood, and adult thorax width. The data were statistically analyzed by Fisher's exact probability test and Mann-Whitney U test by using the software R v2.6.2 [69].

### Pulsed field gel electrophoresis

A portion of the midgut crypts was dissected from a female adult insect, and was homogenized in 50 μl of PBS containing 10 mM EDTA. The homogenate was filtered through a 10 μm nylon mesh, and the filtered suspension was mixed with 1% low melting point agarose. The agarose plug was cut into an appropriate shape and size, and treated with proteinase K at 50°C overnight. After thorough washing in TE buffer, the plug was subjected to digestion with either of the restriction endonucleases *AscI, ApaI, I-CeuI*, or *SmaI*. Pulsed field gel electrophoresis was conducted by using CHEF Mapper XA (Bio-Rad).

## Authors' contributions

YKi, TH, and TF designed and coordinated the experiments. YKi carried out the molecular genetic analyses, histological studies, and fitness measurements. TH performed the statistical analysis and pulsed field gel electrophoresis. NN carried out the phylogenetic analysis of a protein-coding gene and relative rate test. XYM performed the electron microscopy. YKa and TF contributed reagents/materials/analysis tools. YKi and TF wrote the paper. All authors read and approved the final manuscript.

## Supplementary Material

Additional file 1 **Supplementary Table s1.** List of acanthosomatid stinkbug samples used in this study. Click here for file

Additional file 2 **Supplementary Figure s1**. All possible fully-resolved trees of the symbiotic bacteria from the stinkbug genus Acanthosoma. Click here for file
