# Supplementary material for: Host-symbiont co-speciation and reductive genome evolution in gut symbiotic bacteria of acanthosomatid stinkbugs
Source: BMC Biol. 2009 Jan 15;7:2. doi: 10.1186/1741-7007-7-2 (PMC2637841; doi:10.1186/1741-7007-7-2)
Supplement: Additional file 1 — Supplementary Table s1. List of acanthosomatid stinkbug samples used in this study. [file 1741-7007-7-2-S1.pdf]

Additional file 1: Table. Acanthosomatid stinkbug samples used in this study.

Insect species	Collection locality[a]	Collection date	Collector	DNA accession number		
				16S rRNA	groEL	COI
Elasmostethus humeralis	Sapporo, Hokkaido (SP[b])	1 July 1995	S. Kudo	AB368823		
	Higashitsuno, Kochi (HG[b])	13 July 2002	M. Takai	AB368825		
	Tsukuba, Ibaraki (TK[b])	22 October 2002	Y. K	AB368824		AB368840
		14 September 2004 [c]	Y. K			
		11 October 2007 [d]	Y. K			
Elasmostethus nubilus	Kitaibaraki, Ibaraki	14 August 2002	Y. K	AB368822	AB379694	AB368842
	Tsukuba, Ibaraki	14 September 2004 [c]	Y. K			
		11 October 2007 [d]	Y. K			
Elasmostethus brevis	Sapporo, Hokkaido	27 July 1995	S. Kudo	AB368826		AB368841
Lindbergicoris gramineus	Kumakogen, Ehime	22 July 2000	M. Takai	AB368827		AB368843
Elasmucha putoni	Sapporo, Hokkaido	10 June 2003 [d]	S. Kudo			
	Tsukuba, Ibaraki	21 June 2006	T. H	AB368828	AB379695	AB368844
Elasmucha dorsalis	Shintoku, Hokkaido	7 September 2003	S. Kudo	AB368829		AB368845
Elasmucha signoreti	Kumakogen, Ehime	7 July 2002	M. Takai	AB368830		AB368846
Sastragala esakii	Suwa, Nagano (SW[b])	30 October 2001	Y. K	AB368831		
	Tsukuba, Ibaraki (TK[b])	22 October 2002	Y. K	AB368832	AB379696	AB368848
Sastragala scutellata	Higashitsuno, Kochi	13 July 2002	M. Takai	AB368833		AB368847

Table S1. Continued.

Insect species	Collection locality	Collection date	Collector	DNA accession number		
				16S rRNA	groEL	COI
Acanthosoma haemorrhoidale	Sapporo, Hokkaido (SP[b])	29 June 1995	S. Kudo	AB368834		AB368849
	Kumakogen, Ehime (KM[b])	6 July 2002	M. Takai	AB368835		
Acanthosoma labiduroides	Higashitsuno, Kochi	5 July 2002	M. Takai	AB368836		AB368850
Acanthosoma forficula	Higashitsuno, Kochi	21 July 2002	M. Takai	AB368837		AB368851
Acanthosoma denticaudum	Obihiro, Hokkaido	27 August 1995	S. Kudo	AB368838		AB368852
Acanthosoma giganteum	Nitta, Gunma	4 July 2002	E. Hara	AB368839		AB368853

[a] All localities in Japan.

[b] Sample name used in figure 3.

[c] Subjected to transmission electron microscopy.

[d] Subjected to diagnostic PCR analysis.
