# Supplementary material for: Host-symbiont co-speciation and reductive genome evolution in gut symbiotic bacteria of acanthosomatid stinkbugs
Source: BMC Biol. 2009 Jan 15;7:2. doi: 10.1186/1741-7007-7-2 (PMC2637841; doi:10.1186/1741-7007-7-2)
Supplement: Additional file 2 — Supplementary Figure s1. All possible fully-resolved trees of the symbiotic bacteria from the stinkbug genus Acanthosoma. [file 1741-7007-7-2-S2.ppt]

## Slide 1
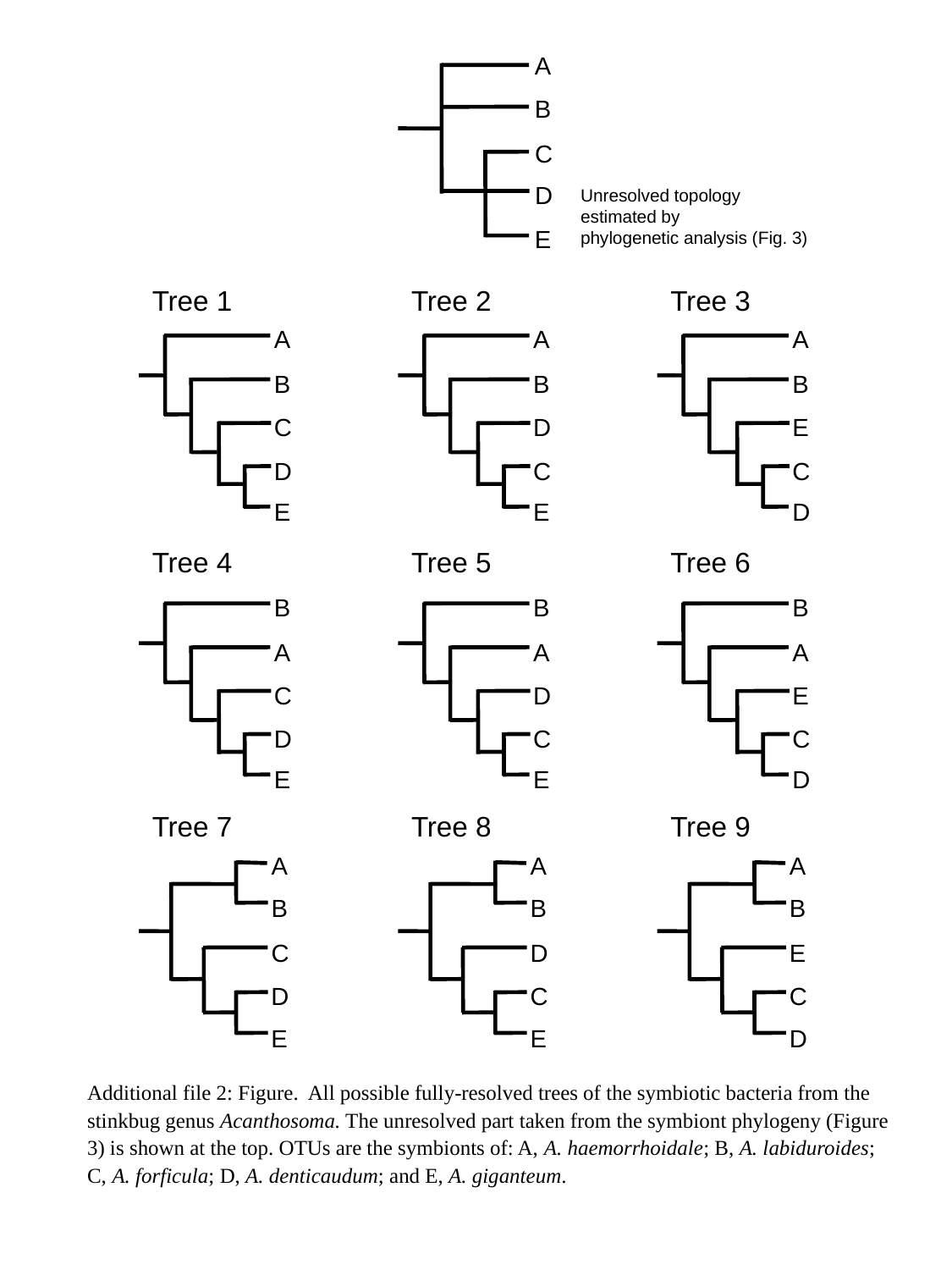

A
B
C
D
E
Unresolved topology
estimated by
phylogenetic analysis (Fig. 3)
Tree 1
Tree 2
Tree 3
A
B
C
D
E
A
B
D
C
E
A
B
E
C
D
Tree 4
Tree 5
Tree 6
B
A
C
D
E
B
A
D
C
E
B
A
E
C
D
Tree 7
Tree 8
Tree 9
A
B
C
D
E
A
B
D
C
E
A
B
E
C
D
Additional file 2: Figure. All possible fully-resolved trees of the symbiotic bacteria from the stinkbug genus Acanthosoma. The unresolved part taken from the symbiont phylogeny (Figure 3) is shown at the top. OTUs are the symbionts of: A, A. haemorrhoidale; B, A. labiduroides; C, A. forficula; D, A. denticaudum; and E, A. giganteum.
